# Putative stem cell markers in cervical squamous cell carcinoma are correlated with poor clinical outcome

**DOI:** 10.1186/s12885-015-1826-4

**Published:** 2015-10-24

**Authors:** Teng Hou, Weijing Zhang, Chongjie Tong, Gallina Kazobinka, Xin Huang, Yongwen Huang, Yanna Zhang

**Affiliations:** 1 State Key Laboratory of Oncology in South China, Sun Yat-sen University Cancer Center, Collaborative Innovation Center for Cancer Medicine, Guangzhou, GD 510060 China; 2 Department of Urology, Union Hospital, Tongji Medical College, Huazhong University of Science and Technology, Wuhan, HB 430022 China

**Keywords:** Cervical squamous cell carcinoma, Cancer stem cell, Prognosis, Chemoresistance, Musashi-1, ALDH1, Sox2, CD49f

## Abstract

**Background:**

The aim of this study was to elucidate the value of putative cancer stem cell markers Musashi-1, ALDH1, Sox2, and CD49f in predicting the prognosis in cervical squamous cell carcinoma (CSCC).

**Methods:**

Real-time PCR and immunohistochemistry staining was performed to examine Musashi-1, ALDH1, Sox2, and CD49f expression in archived specimens of CSCC patients with postoperative chemotherapy. Kaplan–Meier analysis and Cox proportional hazards model were used to assess the prognostic impact of CSC markers for overall survival (OS) and recurrent-free survival (RFS).

**Results:**

The Real-time PCR data showed that the expression of all markers were increased in CSCC tissues compared with in paired normal cervical tissues (*P* < 0.05). The IHC result showed that high expression of Msi1, ALDH1, Sox2, and CD49f was found in 25.7 %, 43.0 %, 62.0 % and 29.0 % CSCC samples, respectively. Moreover, high expression of Msi1 (*P* = 0.033 and *P* = 0.003, respectively), ALDH1 (*P* = 0.015 and *P* = 0.002, respectively), and Sox2 (*P* = 0.005 and *P* = 0.003, respectively), and low expression of CD49f (*P* = 0.027 and *P* = 0.025, respectively) were correlated with poor OS and PFS in CSCC patients. Interestingly, tumors with Msi1_high_/CD49f_low_ expression had the poorest prognosis according to Msi1/CD49f stratification. In multivariate Cox regression analysis, Sox2 expression (*P* = 0.047 and *P* = 0.018, respectively), ALDH1 expression (*P* = 0.013 and *P* = 0.003, respectively), and CD49f expression (*P* = 0.008 and *P* = 0.003, respectively) were independent prognostic markers for both OS and RFS.

**Conclusions:**

Our results suggest that cancer stem cell markers are linked with poor prognosis of CSCC patients.

**Electronic supplementary material:**

The online version of this article (doi:10.1186/s12885-015-1826-4) contains supplementary material, which is available to authorized users.

## Background

Cervical cancer is the second most common gynecologic cancer in developing countries, with over 500,000 new cases and 274,000 deaths annually [1]. Despite advances in surgical capabilities and chemotherapy strategies, a substantial proportion of patients still die from recurrent or chemoresistant disease. Approximately 30–50 % of advanced-stage patients will develop recurrent disease [2]. Even in patients with early-stage disease, 10–20 % will recur locoregionally or have distant metastases following treatment. The reported median overall survival time of patients who recur after radical surgery or radiotherapy are between 7 and 12 months [3]. Adjuvant platinum-based drugs are standard chemotherapy treatment for cervical cancer. Meanwhile, the duration for response to chemotherapy in patients with recurrent diseases remains disappointing.

Currently, there is a lack of biomarkers for predicting the response to chemotherapy in cervical cancer patients.

Cancer stem cells (CSCs) are defined as a small population of cells within a tumor that can self-renew and drive tumorigenesis. Increasing evidence has suggested that CSCs are naturally resistant to chemotherapeutic agents and might be responsible for tumor recurrence following chemotherapy [4]. The potential mechanisms of CSC resistance to chemotherapy include slow cell cycle kinetics, overexpression of DNA repair proteins and multidrug resistance transporters, and protection by hypoxic niches [5].

It is postulated that CSCs may originate from normal stem cells and express the same cell markers as normal stem cells. Such a model has been proposed for cervical CSC markers as well [6]. To date, several potential cervical epithelial stem cell markers including Musashi-1 (Msi1), ALDH1, SOX2, and CD49f have been used to identify cervical CSCs (CCSCs). Musashi-1 is a RNA-binding protein to regulate the proliferation of multipotential stem/progenitor cells and the proliferative activity of tumor cells [7]. This protein may also have roles in cervical carcinogenesis [8]. ALDH1 is a polymorphic enzyme involved in oxidation of aldehydes to carboxylic acids. It has been reported to enhance the self-renewal and differentiation potentials of cervical cancer cells [9]. Sox2 is essential for the formation of many different tissues and organs during embryonic development. Sox2-positive population of cervical cancer cells show characteristics of tumor-initiating cells. Its expression increases the expression of CSC markers, the potential to form tumor spheres, and the tumor initiating capacity of cervical cancer cells [10]. CD49f gene encodes an integral cell-surface protein that has been proposed to play a role in cell adhesion as well as in cell-surface mediated signaling. CD49f has been used as an epithelial stem cell marker and was recently found highly expressed on cervical cancer-initiating cells [11].

As CSCs are thought to be responsible for tumor chemoresistance and recurrence, the evaluation of CSC markers expression in cervical cancer could highlights the mechanisms underlying cervical cancer progression and recurrence. Moreover, to determine the association of CSC markers expression to cervical cancer chemoresponsiveness could help in the development of targeted agents to treat chemoresistant disease. The aims of this work were therefore to evaluate the expression pattern of Msi1, ALDH1, Sox2, and CD49f in cervical cancer tissues and to determine their significance in predicting the prognosis in cervical cancer patients.

## Methods

### Patients and tissue specimens

A total of 179 cervical cancer patients treated between January 2001 and December 2008 were included in the study. The material was retrieved from archival paraffin-embedded surgical samples at Sun Yat-Sen University Cancer Center. In addition, 75 pairs of snap-frozen cervical cancer and normal cervical samples from above patients were collected for Real-time PCR. None of the patients had received chemotherapy or radiotherapy before surgery. After surgery, the patients were treated with adjuvant chemotherapy according to the national guidelines. Platinum-based chemotherapy was initiated within two weeks after surgery and then repeated for four cycles at three-week intervals. In all cases, the diagnoses and grading were peer-reviewed according to the principles laid down in the latest International Federation of Gynecology and Obstetrics criteria [12]. Prior written consent was obtained from all patients and this study was approved by the Research Ethics Committee of Sun Yat-Sen University Cancer Center.

### RNA extraction and real-time PCR

Total RNA from CSCC and paired normal cervical tissues was extracted using Trizol reagent (Invitrogen, Carlsbad, CA, USA) according to the manufacturer’s instructions. The extracted RNA was pretreated with RNase-free DNase, and 2 μg RNA from each sample was used for cDNA synthesis with random hexamers. Real-time PCR was performed using the Applied Biosystems 7500 Sequence Detection system. The primers used are as follows: Msi1, forward, 5’- GAGGGTTCGGGTTTGTCACG-3’, reverse, 5’-GGCGACATCACCTCCTTTGG-3’; ALDH1, forward, 5’-GTTAGCTGATGCCGACTT GG-3’ , reverse, 5’-CCCACT CTCAATGAGGTCAAG-3’; Sox2, forward, 5’- GCTGTATGGCTGCTGCACTTC-3’, reverse, 5’-GCACACGCACCCAGCACT GT-3’; CD49f, forward, 5’- ATGGAG GAAACCCTGTGGCT-3’, reverse, 5’- ACGAGAGCTTGG CTCTTG GA-3’; GAPDH, forward, 5’-AGAAGGCTGGGCTCATTTG-3’, reverse, 5’-AGGGGCCATCCA CAGTCTTC-3’. The expression level of CSC markers mRNA was calculated using a ratio of CSC markers mRNA to GAPDH mRNA.

### Immunohistochemical (IHC) staining

Immunostaining was performed on paraffin-embedded 4 μm sections and mounted on poly-L-lysine-coated slides. The sections were baked at 65 °C for 30 minutes, then deparaffinized in xylene and rehydrated. Antigen retrieval was performed by submerging the sections into a 10 μmol/L citrate buffer solution (pH 6.0) for 10 minutes in a microwave oven. The slides were then treated with 3 % hydrogen peroxide in methanol to quench the endogenous peroxidase activity, followed by incubation with 1 % fish skin gelatin to block the nonspecific staining. Tissue sections were incubated overnight with monoclonal rabbit antibody against Msi1 (Abcam, Cambridge, USA; 1:200), monoclonal rabbit antibody against ALDH1 (Abcam, Cambridge, USA; 1:200), monoclonal mouse antibody against Sox2 (Abcam, Cambridge, USA; 1:200), and monoclonal rabbit antibody against CD49f (Abcam, Cambridge, USA; 1:200). After washing, the sections were incubated with prediluted secondary antibody (Abcam, Cambridge, USA), followed by further incubation with 3,3-diaminobenzidine tetrahydrochloride (DAB). Finally, the slides were counterstained with hematoxylin and mounted in an aqueous mounting medium.

For negative controls, primary antibodies were replaced with normal serum.

### IHC score

Immunostaining was separately evaluated by two independent pathologists who were blinded as to the patients. Expression of the four CCSC markers was analyzed by an individual labeling score considering percent and staining intensity of positive cells. Intensity of stained cells was graded semi-quantitatively into four levels: 0 (no staining); 1 (weak staining = light yellow); 2 (moderate staining = yellow brown) and 3 (strong staining = brown); and the percentage was scored as: 0, negative; 1, 10 % or less; 2, 11 % to 50 %; 3, 51 % to 80 %; or 4, 80 % or more positive cells. Intensity and fraction of positive cell scores were multiplied for each marker and thus the scoring system was defined as low expression for scores of 0–3, and as high expression for scores of 4–12.

### Statistical analysis

All statistical analyses were carried out using SPSS (version 16.0, SPSS Inc, Chicago, USA) statistical software. The overall survival (OS) and recurrence-free survival (RFS) were calculated as the time from the date of primary surgery to the date of first death or recurrence. Survival curves were plotted using the method of Kaplan-Meier, and the log-rank test was used to determine statistical differences between life tables. The correlations between clinicopathologic characteristics and recurrence were analyzed using the χ^2^ test. Univariate and multivariate analysis were applied to assess the independent predictive significance of variables on RFS. *P* < 0.05 in all cases was considered statistically significant.

## Results

### Clinical results and tumor recurrent

Patients’ clinicopathological characteristics were shown in Table 1. In total, 179 cervical cancer patients with up to stage IIB were investigated. The median follow-up period of the patients was 48.8 months (range 1.6–60.0 months). After postoperative chemotherapy, 22 patients had recurrent disease.

**Table 1:** Clinicopathological characteristics

Characteristic	No. of Patients	Percent
Age (y)		
≤40	108	60.3
>40	71	39.7
FIGO Stage		
IB1	96	53.6
IB2	40	22.3
IIA1	29	16.2
IIA2	11	6.2
IIB	3	1.7
Differentiation		
Well	7	3.9
Moderately	65	36.3
Poorly	107	59.8
Tumor Size		
≤4 cm	120	67.0
>4 cm	59	33.0
Total No. of Patients	179	100

### Patterns of expression

**Fig. 1:**
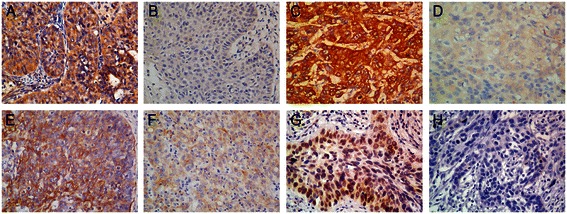
The expression of Msi1, ALDH1, CD49f, and Sox2 in cervical squamous cell carcinoma by immunohistochemistry. **a** and **b**, high and low immunoreactivity of Msi1. **c** and **d**, high and low immunoreactivity of ALDH1. **e** and **f**, high and low immunoreactivity of CD49f. **g** and **h**, high and low immunoreactivity of Sox2. (original magnification 400**×**)

**Fig. 2:**
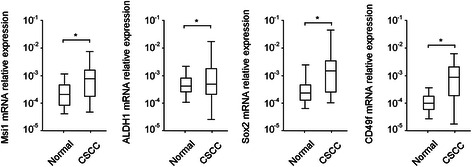
Relative mRNA expression of Msi1, ALDH1, Sox2, and CD49f in normal cervical and CSCC tissues. * *P* < 0.05

To investigate the potential roles of CSC markers in the development of cervical cancer, we determined the expression of Msi1, ALDH1, Sox2 and CD49f in 179 paraffin-embedded cervical carcinoma samples by immunohistochemistry. Msi1 immunoreactivity was both cytoplasmic and nuclear and high expression was observed in 46 cases (25.6 %). ALDH1 immunoreactivity was cytoplasmic and high expression was observed in 77 cases (43.0 %). Sox2 immunoreactivity was nuclear and high expression was observed in 111 cases (62.0 %). CD49f immunoreactivity was both membranous and cytoplasmic and high expression was observed in 52 cases (29.0 %). The representative immunostaining of Msi1 (Fig. 1a–b), ALDH1 (Fig. 1c–d), Sox2 (Fig. 1e–f) and CD49f (Fig. 1g–h) were shown in Fig. 1. Moreover, Real-time PCR showed that the expression level of all CSC markers was increased in CSCC compared with that in normal cervical tissues (Fig. 2).

### Associations between Msi1, ALDH1, Sox2 and CD49f expression and clinicopathological features

The expression of CCSC markers was evaluated in relation to clinicopathological variables for cervical cancer. Msi1, ALDH1, and Sox2 expression were positively correlated with tumor recurrence (*P* = 0.005, *P* = 0.003, and *P* = 0.003, respectively), while CD49f expression was negatively correlated with tumor recurrence (*P* = 0.028) (Table 2). Msi1, ALDH1 and CD49f expression was not associated with any of the clinicopathological variables, while only Sox2 expression was significantly associated with tumor size (*P* = 0.010). (Additional file 1: Table S1).

**Table 2:** Association of cancer stem cell markers expression and tumor recurrence

	No. of patients (%)		*p*
	Recurrence	No Recurrence	
FIGO Stage			0.008
IB1	6(27.3)	90(57.3)	
>IB1	16(72.7)	67(42.7)	
Differentiation			0.205
Grade 1, 2	6(27.3)	65(41.4)	
Grade 3	16(72.7)	92(58.6)	
Timor Size			0.069
≤4 cm	11(50.0)	109(69.4)	
>4 cm	11(50.0)	48(30.6)	
Msi1			0.005
High	11(50.0)	35(22.3)	
Low	11(50.0)	122(79.7)	
ALDH1			0.003
High	16(72.7)	61(38.9)	
Low	6(27.3)	96(61.1)	
CD49f			0.028
High	2(9.1)	50(31.8)	
Low	20(90.9)	107(68.2)	
Sox2			0.003
High	20(90.9)	91(58.0)	
Low	2(9.1)	66(42.0)	

### Associations between Msi1, ALDH1, Sox2 and CD49f expression and clinical prognosis

**Fig. 3:**
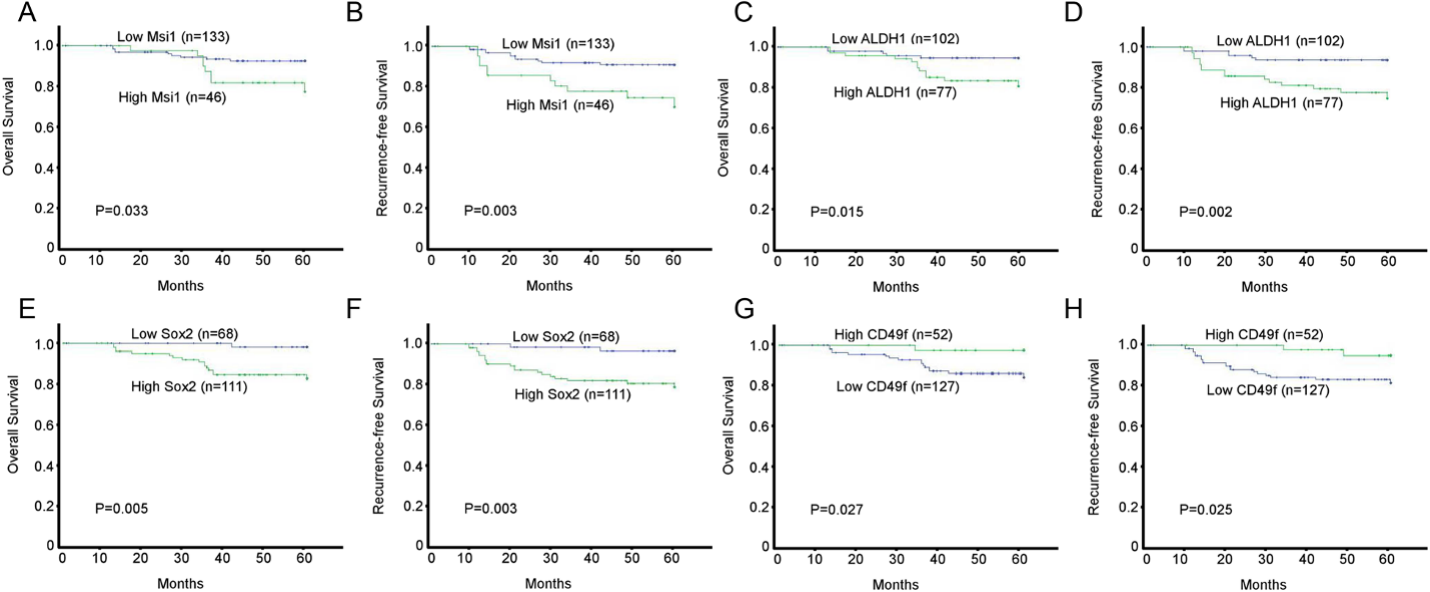
Kaplan–Meier estimated of overall survival and recurrence-free survival according to putative stem cell markers expression in CSCC patients. **a** Overall survival and **b** recurrence-free survival time differences in patients with low vs high expression of Msi1. **c** Overall survival and **d** recurrence-free survival time differences in patients with low vs high expression of ALDH1. **e** Overall survival and **f** recurrence-free survival time differences in patients with low vs high expression of Sox2. **g** Overall survival and **h** recurrence-free survival time differences in patients with low vs high expression of CD49f

**Fig. 4:**
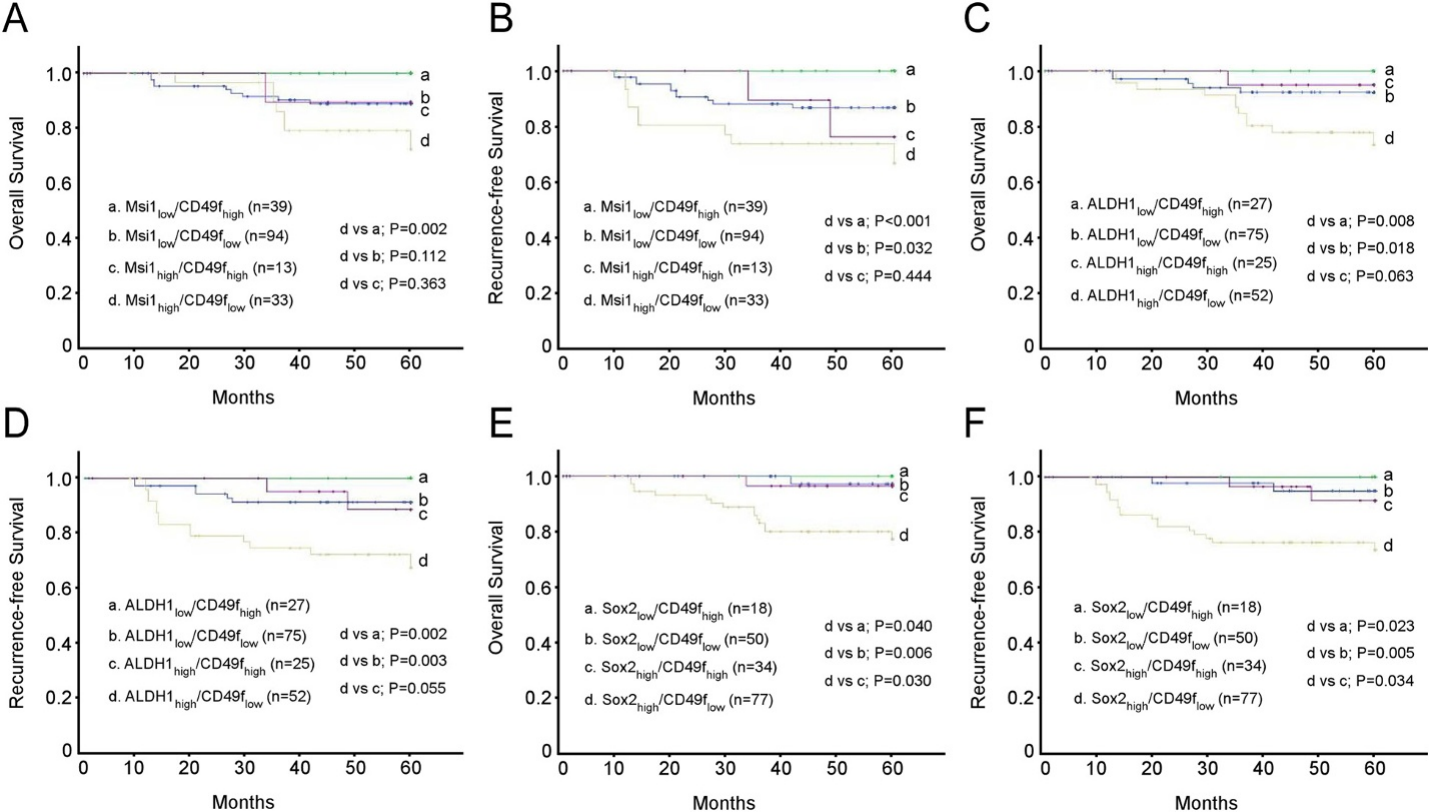
Kaplan–Meier estimates of overall survival and recurrence-free survival according to combinations of putative stem cell markers. **a** Overall survival and **b** recurrence-free survival according to Msi1 and CD49f expression. **c** Overall survival and **d** recurrence-free survival according to ALDH1 and CD49f expression. **e** Overall survival and **f** recurrence-free survival according to Sox2 and CD49f expression

To examine the prognostic value of CSCs markers in cervical cancer, we evaluated the correlation between Msi1, ALDH1, Sox2 and CD49f expression and prognosis in CSCC patients. Notably, high expression of Msi1 (*P* = 0.033 and *P* = 0.003, Fig. 3a and b), ALDH1 (*P* = 0.015 and *P* = 0.002, Fig. 3c and d), and Sox2 (*P* = 0.005 and *P* = 0.003, Fig. 3e and f), and low expression of CD49f (*P* = 0.027 and *P* = 0.025, Fig. 3a and d) were correlated with poor OS and RFS in all the 179 CSCC patients who received postoperative adjuvant chemotherapy. Moreover, double stratification analysis of OS and RFS according to Msi1/CD49f, ALDH1/CD49f, and Sox2/CD49f were performed and shown in Fig. 4. Tumors with Msi1_high_/CD49f_low_, ALDH1_high_/CD49f_low_, and Sox2_high_/CD49f_low_ expression had a poorer prognosis compared with those with Msi1_low_/CD49f_high_ (*P* = 0.002 and *P* < 0.001, Fig. 4a and b), ALDH1_low_/CD49f_high_ (*P* = 0.008 and *P* = 0.002, Fig. 4c and d), and Sox2_low_/CD49f_low_ expression (*P* = 0.006 and *P* = 0.005, Fig. 4e and f), respectively.

Univariate and multivariate Cox proportional hazards regressions analysis were also applied. Univariate analysis showed that FIGO stage, tumor size, and Msi1 expression were significantly correlated with both OS and RFS. The ALDH1 expression, Sox2 expression, and CD49f expression were significantly associated with RFS (data not shown). Multivariate analysis showed that FIGO stage > IB1 (*P* = 0.002 and *P* = 0.005, respectively), high Sox2 expression (*P* = 0.047 and *P* = 0.018, respectively), high ALDH1 expression (*P* = 0.013 and *P* = 0.003, respectively), and low CD49f expression (*P* = 0.008 and *P* = 0.003, respectively) were independent prognostic factors for both OS and RFS (Table 3).

**Table 3:** Multivariate analysis of overall and recurrence–free survival

Prognostic variables	Overall survival		Recurrence–free survival	
	Hazard ratio (95 % CI)	*P*	Hazard ratio (95 % CI)	*P*
Age (>40 vs ≤40)				
FIGO Stage (>IB1 vs IB1)	7.467 (2.107–26.460)	0.002	3.938 (1.524–10.197)	0.005
Differentiation (Grade 3 vs 1/2)				
Timor Size (>4 cm vs ≤4 cm)				
Msi1 (high vs low)				
ALDH1 (high vs low)	3.805 (1.331–10.879)	0.013	4.261 (1.655–10.968)	0.003
CD49f (high vs low)	0. 064 (0.008–0.492)	0.008	0.108 (0.025–0.470)	0.003
Sox2 (high vs low)	8.650 (1.141–65.603)	0.047	5.834 (1.353–25.163)	0.018

## Discussion

CSCs are generally thought to arise from normal stem cells that have developed genetic mutations over time. CCSCs could arise from Müllerian duct-derived cervical stem cells located in the basal layer of the ectocervical squamous or endocervical columnar epithelium [13]. These cells have been proposed to drive the resistance to chemotherapy through the expression of drug efflux pumps in normal stem cells from which they were derived [14]. Therefore, the identification of the CSCs subpopulation of tumor cells may offer new directions for the treatment of cervical cancer. In the present work, we provide the first link between the expression of four putative CCSC markers and the clinical outcome in cervical cancer patients.

Previous studies have demonstrated that CSCs are responsible for the high rate of chemoresistance and tumor relapse. The possible mechanisms of chemoresistance include high expression levels of adenosine triphosphate-binding cassette transporters, low self-renewal rate, an active DNA repair capacity, and activated Wnt/β-catenin and Notch signaling [15]. Clinical and experimental studies have provided evidence of the relationship between different CSCs phenotypes and resistance to chemotherapeutic drugs. Manohar et al. proposed that overexpression of MYCN, the most widely characterized gene associated with poor outcome of neuroblastoma, resulted in increased drug resistance and expression of MRP1, suggesting that MRP1 may be a MYCN target gene involved in the drug-resistance phenotype of neuroblastoma [16]. Wang et al. reported that overexpression of caveolin-1 enhanced chemoresistance of breast CSCs, which could be prevented by downregulation of the β-catenin/ABCG2 pathway [17]. Recent studies also implicated CSCs in cervical cancer chemoresistance [18], while there is lack of data concerning the relevance of the expression of CSC markers with clinical prognosis and chemosensitivity in cervical cancer. Our findings suggest that the expression of CSC markers Msi1, ALDH1, Sox2, and CD49f were increased in CSCC tissues and that their expression are linked to clinical survival in cervical cancer patients undergoing postoperative chemotherapy, suggesting an important role of these markers in predicting clinical prognosis in cervical cancer. Surprisingly, we found that the expression of CSC markers showed no association with age, tumor stage, tumor size, or tumor differentiation, while only Sox2 expression was significantly correlated with tumor size. The results may be due to small group size.

ALDH1 has long been thought to be a CSC marker and has also been described to be associated with tumor progression and recurrence [19]. Many recent studies investigating the ALDH1 gene or protein report its impact on the clinical outcome of patients with malignancies. Dylla et al. reported that the inherent chemotherapeutic resistance mechanism of colorectal CSCs includes ALDH1 enzymatic activity [20]. Alamgeer et al. described ALDH1 as a useful predictor of chemoresistance in locally advanced breast cancer [21]. Our present investigation found that high ALDH1 expression was an independent predictor of recurrence and overall survival in CSCC patients. Among patients with high ALDH1 expression, 21 % had recurrent cancer, whereas only 6 % patients with low ALDH1 expression did. Our study is supported by Deng et al., who demonstrated that high residual ALDH1 expression after radiochemotherapy significantly predicted tumor metastasis or recurrence [22]. The same finding was also applied for another potential CCSC marker Sox2. It has recently been linked with enhanced chemoresistance and tumorigenecity of gastric cancer derived cancer stem-like cells [23]. Conversely, suppression of Sox2 can impair the chemosensitivity and stemness of cancer cells [24]. In the present study, we found that up-regulation of Sox2 predict poor overall and recurrence-free survival in CSCC patients. Among patients with high Sox2 expression, 18 % had recurrent cancer, whereas only 3 % patients with low Sox2 expression did, implicating a correlation of Sox2 expression and cervical cancer progression. Relatively fewer studies have evaluated the roles of Msi1 in chemoresistance. Li et al. identified a population of chemoresistant SP cells from gastric cancer cell line and observed a high expression level of Msi1 in these cells [25]. Johannessen et al. demonstrated that the expression of Msi1 was downregulated through inhibition of PI3K/AKT pathway, which is involved in treatment resistance in glioblastomas [26]. In this study, we document a poorer overall and recurrence-free survival time with high expression of Msi1 in univariate but not multivariable survival time analysis, indicating that the poor prognostic role of Msi1 may be secondary to their association with other established prognostic criteria. Altogether, these studies suggest that increased ALDH1, Sox2, and Msi1 expression are related to the progression and may be related to chemoresistance of CSCC.

Interestingly, we found that CD49f expression was increased in CSCC tissues while high expression of CD49f was associated with favourable prognosis of patients. The results suggested that CD49f expression was linked with reduced chemoresistance in cervical cancer. CD49f has been reported to play a critical role in CSC maintenance and in the attachment of tumor cells to laminin [27]. The unexpected finding that CD49f expression was correlated with better clinical outcome suggests that not all CSC markers characterise the chemoresistant cell population and that the prognostic role of CD49f might be tissue specific and diverse in different cancers. Another explanation is that the expression of CD49f may be decreased in the invasive area of cancer tissues, which has already been reported for other cell adhesion molecules, and which has been regarded as a key event in epithelial mesenchymal transition [28].

An important finding emerges from the present investigation is that tumors with high Msi1 and low CD49f expression had the poorest prognosis, whereas tumors with lack of Msi1 and CD49f overexpression had the best prognosis. Double stratification analysis was also performed according to ALDH1/CD49f, and Sox2/CD49f, and the results showed that ALDH1_high_/CD49f_low_ and Sox2_high_/CD49f_low_ population predict poor recurrent-free prognosis in CSCC patients. The results imply the importance of such a sub-population in predicting the prognosis and chemosensitivity of cervical cancer patients. Targeting this cell population with novel pharmaceutical agents may prove to be effective for the treatment of chemoresistant cervical tumors.

## Conclusions

In conclusion, our study provided the first clinical evidence that CSC markers are associated with the clinical prognosis of CSCC patients. High expression of Msi1, ALDH1, and Sox2, and low expression of CD49f predict poor prognosis for cervical cancer patients receiving postoperative chemotherapy. Moreover, double stratification analysis showed that Msi1_high_/CD49f_low_ subgroup had the poorest RFS. Our findings may be of great value in the development of personalized therapies for patients with cervical cancer.

## Additional file

Additional file 1: Table S1. Correlation of cancer stem cell markers with histopathological variables. (DOCX 15 kb)
