# Supplementary material for: Putative stem cell markers in cervical squamous cell carcinoma are correlated with poor clinical outcome
Source: BMC Cancer. 2015 Oct 24;15:785. doi: 10.1186/s12885-015-1826-4 (PMC4619529; doi:10.1186/s12885-015-1826-4)
Supplement: Additional file 1: Table S1. — Correlation of cancer stem cell markers with histopathological variables. (DOCX 15 kb) [file 12885_2015_1826_MOESM1_ESM.docx]

Additional file 1: Table S1. Correlation of cancer stem cell markers with histopathological variables

| Charecrestic | No. | Msi1  High N (%) | *P* | ALDH1  High N (%) | *P* | Sox2  High N (%) | *P* | CD49f  High N (%) | *p* |
| --- | --- | --- | --- | --- | --- | --- | --- | --- | --- |
| Age (y) | 0.540 0.867 0.746 0.644 | | | | | | | | |
| ≤40 | 108 26(24.1) 47(43.5) 68(62.9) 30(27.8) | | | | | | | | |
| >40 | 71 20(28.2) 30(42.2) 43(60.5) 22(40.0) | | | | | | | | |
| FIGO Stage | 0.567 0.929 0.636 0.340 | | | | | | | | |
| IB1 | 96 23(24.0) 41(42.7) 58(60.4) 25(26.0) | | | | | | | | |
| >IB1 | 83 23(27.7) 36(43.4) 53(63.9) 27(32.5) | | | | | | | | |
| Differentiation | 0.600 0.765 0.605 0.099 | | | | | | | | |
| Grade 1/2 | 72 17(23.6) 30(41.7) 43(59.7) 16(22.2) | | | | | | | | |
| Grade 3 | 107 29(27.1) 47(43.9) 68(63.6) 36(33.6) | | | | | | | | |
| Timor Size | 0.953 0.071 **0.010** 0.690 | | | | | | | | |
| ≤4cm | 120 31(25.8) 46(38.3) 67(55.8) 36(30.0) | | | | | | | | |
| >4cm | 59 15(25.4) 31(52.5) 44(74.6) 16(27.1) | | | | | | | | |
| Total No. of  Patients | 179 46 77 111 52 | | | | | | | | |
